# The De La Rue Scheme of Medical Service

**Published:** 1920-07-31

**Authors:** 


### THE EDITOR'S LETTER-BOX.

**Correspondence on all subjects is invited, but we cannot in any way be responsible for the opinions expressed by our correspondents, who must give their name and address as a guarantee of good faith, but not necessarily for publication. Correspondents are reminded that brevity of style and conciseness of statement greatly facilitate early insertion.**

### The De La Rue Scheme of Medical Service.

*To the Editor of* The Hospital.

Sir,—I am sure that anyone interested in the provision of efficient medical advice and treatment for persons of slender or moderate incomes are further indebted to Mr. Stuart De La Rue for his letter published in your issue of the 24th inst. Mr. De La Rue's explanations certainly meet some of my criticisms, and it is so far satisfactory to know that the absence of the family practitioner from the scheme, as first described, does not mean that he is to be replaced by the "company's medical adviser." There is a real difficulty to be met, and the company's proposals are a sincere effort to meet it. But at the best they apply to a limited field, and the position will never be satisfactorily regulated until the medical profession faces the situation and adopts a plan capable of general application. The prospect of every considerable business firm setting up its own list of specially approved consultants is neither an inviting nor a dignified one, and there are certainly some members of the profession who would refuse to take part in an enterprise that cannot be freed from the reproach of personal advertisement. The nursing-home proposal being "in the embryonic state" may be left for the present, but I may express the hope that, before an attempt is made to bring it to maturity, counsel will be sought from some authority competent to speak for the whole of the medical profession. Mr. De La Rue does not tell us how he obtained an assurance of "whole-hearted" professional support for his original scheme, and I may be excused for continuing to think that the pledge, whatever its source, possesses merely a rhetorical and restricted value.

"X²" reads the De La Rue plan as a "charitable" organisation with a board of managers, a medical staff, and a medical and surgical registrar to "weed out" the cases. And yet he tells us that any beneficiary under the scheme will return to his home and work with the pious consolation that "he has not had to sponge on public charity." "X²'s" suggestion of the likeness of the De La Rue scheme to that of a voluntary hospital will, I am sure, be repudiated by its authors, and his analogy is false in at least the particulars. The doctors on a hospital staff receive no pecuniary recognition of their work, and the patients pay no professional fees. In these circumstances, the publication of the list of the doctors concerned, and the exercise of choice by the patients, take on an entirely different complexion. The selected practitioner is not the "firm's" adviser; he is the adviser of the individual patient, and from the individual patient he receives his fee. In "X²'s" views on "mutual" benefit, medical directories, insurance societies, official reports, and professional advertisements I will not pretend to find any points of interest. He is, of course, entitled to enjoy them, and I make no attempt to disturb his belief that they have some value in the present discussion.—I am, Sir,

The Writer of the Original Article.

July 24, 1920.

